# Anemia Treatment, Hemoglobin Variability, and Clinical Events in Patients With Nondialysis-Dependent CKD in Japan

**DOI:** 10.34067/KID.0000000000000204

**Published:** 2023-07-10

**Authors:** Takahiro Kuragano, Suguru Okami, Sachiko Tanaka-Mizuno, Hidetoshi Uenaka, Takeshi Kimura, Yosuke Ishida, Kanae Yoshikawa-Ryan, Glen James, Takanori Hayasaki

**Affiliations:** 1 Division of Kidney and Dialysis, Department of Internal Medicine, Nishinomiya, Hyogo Medical University, Hyogo, Japan; 2 Medical Affairs & Pharmacovigilance, Kita-ku, Bayer Yakuhin, Ltd., Osaka, Japan; 3 Graduate School of Medicine and School of Public Health, Sakyo-ku, Kyoto University, Kyoto, Japan; 4 Research and Analytics Department, Nakagyo-ku, Real World Data Co. Ltd., Kyoto, Japan; 5 Integrated Evidence Generation & Business Innovation, Bayer AG, Reading, United Kingdom

**Keywords:** anemia, cardiovascular events, CKD, clinical epidemiology, epidemiology and outcomes, mortality risk, renal failure

## Abstract

**Key Points:**

- This large, contemporary study reports the management of anemia in a real-world cohort of patients with nondialysis-dependent CKD from multifaceted aspects.
- This study highlights the suboptimal and heterogeneous treatment of anemia in clinical practice.
- The findings also underscore the importance of maintaining a stable hemoglobin concentration within the target range to reduce the risk of mortality and morbidity.

**Background:**

Anemia management in patients with nondialysis-dependent CKD has attracted attention with the introduction of novel therapeutic agents; however, few studies have provided comprehensive epidemiologic information.

**Methods:**

A retrospective cohort study was conducted in adult patients with stage ≥3a nondialysis-dependent CKD and hemoglobin (Hb) <11 g/dl (January 2013–November 2021; *N*=26,626) to assess longitudinal treatment patterns, Hb, and iron parameters (ferritin and transferrin saturation) for anemia management. Time-dependent Cox proportional hazard models were applied to assess the risk of clinical events, including death, cardiovascular events, dialysis introduction, and red blood cell transfusion, associated with temporal fluctuation patterns of Hb levels.

**Results:**

The cumulative incidence of anemia treatment initiation within 12 months was 37.1%, including erythropoiesis-stimulating agents 26.5%, iron oral 16.8%, iron intravenous 5.1%, and hypoxia-inducible factor prolyl hydroxylase inhibitor 0.2%. The mean (±SD) Hb levels were improved from 9.9±1.2 to 10.9±1.6 g/dl at 12 months. Despite erythropoiesis-stimulating agents or hypoxia-inducible factor prolyl hydroxylase inhibitor therapy, 30.1% of patients remained Hb <10 g/dl. The risks of premature death, cardiovascular events, dialysis introduction, and red blood cell transfusion were significantly higher in groups with consistently low Hb or low-amplitude Hb fluctuation around the lower limit of target Hb range than in patients with target Hb range (*P* < 0.05). Similarly, significantly higher risks for dialysis introduction and red blood cell transfusion were associated with high-amplitude Hb fluctuation across target Hb range were observed.

**Conclusions:**

The findings underscore the importance of stable Hb control within the target range to reduce the mortality and morbidity risks in patients with nondialysis-dependent CKD while highlighting the suboptimal and heterogeneous treatment of anemia in clinical practice.

## Graphical abstract

**Figure:**
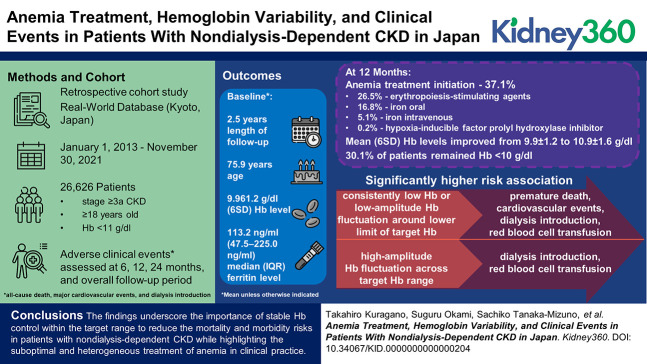

## Introduction

Anemia is a common complication of CKD^1^ and causes numerous unfavorable effects on patients' prognoses and quality of life.^2,3^ Notably higher frequencies of anemia have been reported in patients with advanced kidney disease.^1,4^ In Japan, the prevalence of anemia in a nationwide cross-sectional study was 40% and 60% in patients with CKD stage 4 and stage 5, respectively, on the basis of the Japanese Society of Dialysis Therapy criteria.^5,6^ The pathophysiology of anemia associated with renal failure is often caused by insufficient erythropoiesis and/or iron deficiency (absolute or functional), rather than increased rates of erythrocyte loss or destruction.^1^

The established treatment options for anemia include iron supplementation, erythropoiesis-stimulating agents (ESAs), and red blood cell transfusion.^7^ More recently, hypoxia-inducible factor prolyl hydroxylase inhibitors (HIF-PHIs) have been developed as a novel therapeutic option for renal anemia.^1,8^ However, the optimal treatment algorithm remains controversial, including the trigger of treatment initiation, choice of medication, and the target range of hemoglobin (Hb) and iron parameters for anemia management.^9–12^ Current clinical guidelines recommend a target range of Hb level for the management of anemia, whereas in reality, differences in their recommendations have been observed across geographic regions.^3,13,14^

Anemia management in patients with nondialysis-dependent CKD (NDD-CKD) has recently attracted attention, with the introduction of novel therapeutic agents, and an increasing amount of research has been carried out on this topic. A recent study from CKDopps reported longitudinal anemia treatment patterns in the United States, Germany, and Brazil.^15^ This study alluded to the low initiation rate of anemia treatment in routine clinical care. Similarly, a cross-sectional study of anemia treatment among Japanese patients with CKD was reported but was lacking in longitudinal treatment information, Hb management, and clinical outcomes.^16^ Given the reported geographical variation in treatment patterns,^15^ predisposing clinical factors, and guidelines, it is important to evaluate contemporary and comprehensive aspects of anemia management in patients with NDD-CKD across various regions. Furthermore, although increased risks of adverse clinical events associated with Hb fluctuation were reported in hemodialysis patients,^17^ there is limited information in patients with NDD-CKD regarding various adverse clinical events.^18,19^

The aim of this study was to investigate the longitudinal practice patterns of anemia treatment and the management of Hb and iron parameters, as well as the clinical outcomes in patients with NDD-CKD to provide novel and comprehensive epidemiologic information on anemia management in patients with NDD-CKD. Furthermore, we assessed the associated risk between temporal fluctuations in Hb levels and adverse clinical events, among a broad range of patients with NDD-CKD under continuous medical care in real-world clinical settings.

## Methods

### Study Design, Data Source, and Patient Selection

This study was a retrospective cohort study using a nationwide hospital database in Japan. The study period was from January 1, 2013, to November 30, 2021. The data used in this study were extracted from the Real-World Database (RWD database) maintained by the Health, Clinical, and Education Information Evaluation Institute, with support by Real World Data Co., Ltd. (Kyoto, Japan). This database comprises electronical medical records collected from >200 medical institutions covering most geographic regions and all age groups in Japan. This database includes extensive data on prescriptions, examinations, laboratory data, and hospital diagnoses on the basis of the International Classification of Diseases 10th Revision codes. As of October 2021, the RWD database included medical records from >23 million patients.

We selected patients with stage ≥3a CKD, aged 18 years or older, and with at least one recorded blood test result of Hb <11 g/dl from January 1, 2014, to April 30, 2021. Subsequently, patients already receiving any anemia treatment (*i.e.*, ESA, iron, or HIF-PHI), patients already on dialysis, patients who had a history of major bleeding within 30 days, patients who had malignant tumors and were on chemotherapy and/or radiation therapy, and patients who had hematologic diseases requiring blood transfusion ≥3 times within 6 months were excluded. We also excluded patients without medical records for 6 months before the index date to ensure a sufficient period to collect patients' background information. The index date was set as the date with the first recorded Hb <11 g/dl after confirmation of CKD. Patients were followed up until the time of death, emigration from the dataset, or the end of the study period, whichever came first.

### Clinical Variables and Outcomes

We collected information on demographics, medications, and laboratory data on the basis of the information recorded in the 6 months before the index date. The medical history and comorbidities were retrieved up to 12 months before the index date. The comorbidities collected in this study can be found in Supplemental Table 1. CKD stages (G3a–G5) were categorized on the basis of eGFR using CKD-Modification of Diet in Renal Disease modified for Japanese.^20^ The patterns of anemia treatment, including their initiation and discontinuation, were assessed during the follow-up period. Anemia treatment included any of ESA, iron oral, iron intravenous (iv), or HIF-PHI. Hb levels and iron parameters, including transferrin saturation (TSAT) and ferritin levels, were collected at baseline and during the follow-up period. The occurrence of adverse clinical events was assessed at 6, 12, and 24 months and in the overall follow-up period. The clinical events included all-cause death, major cardiovascular events (myocardial infarction, unstable angina pectoris, stroke, or hospitalization for heart failure), and dialysis introduction. Red blood cell transfusion was collected as an anemia-related clinical event. Detailed definitions of these clinical events are listed in Supplemental Table 2.

### Statistical Analyses

Continuous variables were reported as mean, SD, median, and interquartile range (IQR). Frequency and percentages were used to document categorical measures of interest. Missing data were not imputed. The incidence of anemia treatment initiation was calculated using the cumulative incidence function, considering death as a competitive risk. The longitudinal patterns of anemia treatment, including combinations of different treatment types, were summarized at every 3-month period after the initiation of anemia treatment. Sankey diagrams were computed to show the longitudinal changes in the treatment patterns. The cumulative incidence of treatment discontinuation was assessed in patients who initiated any anemia treatment. For this analysis, treatment discontinuation was defined as not being prescribed with the same treatment after 30 days following the end of the last treatment episode. The cumulative incidences of clinical events were reported with 95% confidence intervals (95% CI). Statistical analyses were performed in the overall patient population and subgroups categorized on the basis of Hb (<10 or ≥10 g/dl), ferritin (<100 or ≥100 ng/ml), TSAT (<20 or ≥20%), and CKD stages (stage 3 or stage 4–5).

Hb levels and iron parameters were summarized at baseline and every 3-month period until 12 months in the follow-up period. Furthermore, the Hb levels and iron parameters were summarized at the timing of treatment initiation and every 3 months thereafter, until 12 months. The Anderson and Gill model^21^ was applied to assess the risk of temporal fluctuation patterns in Hb levels associated with a risk of clinical events. The Hb fluctuation patterns were categorized into six groups on the basis of the previous literature^17,18^: within the target Hb range (target), consistently below the target (low), consistently above the target (high), low-amplitude fluctuation around the upper limit of the target, low-amplitude fluctuation around the lower limit of the target, and high-amplitude fluctuation across the target range (HA). The target Hb range was set as Hb 11–13 g/dl on the basis of the Japanese guidelines.^13,14^ The sensitivity analysis was performed using the target Hb range of 11–12 g/dl. The model was adjusted by Hb fluctuation patterns, the use of anemia treatment, and ferritin category as time-dependent covariates and clinically relevant time-independent covariates. Hazard ratios and 95% CI with *P*-values < 0.05 were considered statistically significant. The detailed method for constructing the time-dependent model is explained in the Supplemental Materials.

Statistical analyses were performed using the SAS software, version 9.4 (SAS Institute Inc., Cary, NC). For the generation of Sankey diagrams, R version 4.2.1 (ggalluvial package) was used. This study followed the Strengthening the Reporting of Observational Studies in Epidemiology statement,^22^ which is elaborated in Supplemental Table 3.

### Ethics Statement

Because patient records were already anonymized and deidentified, informed consent was waived. The use of deidentified data was in accordance with local regulations. The study conduct adheres to the Declaration of Helsinki. This study was reviewed and approved by an independent ethics committee (MINS: MINS-REC-220216).

## Results

### Patient Characteristics

**Figure 1.**
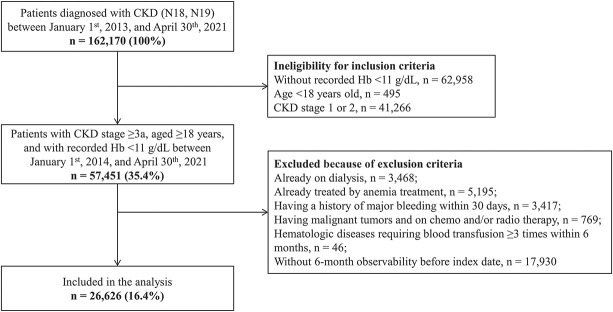
**Flow diagram of patient inclusion in this study.** Hb, hemoglobin.

Of a total of 162,170 patients diagnosed with CKD, we identified 57,451 (35.4%) adult patients with CKD stage ≥3a and recorded Hb <11 g/dl. After applying exclusion criteria, a total of 26,626 (16.4%) patients were included in the analysis (Figure 1). The mean length of follow-up was 2.5 years.

Table 1 shows the baseline characteristics of the overall patient population and subgroups categorized by Hb level. The mean age was 75.9 years, and 37.8% were female patients. The mean (±SD) Hb level and the median (IQR) ferritin level were 9.9±1.2 g/dl and 113.2 ng/ml (47.5–225.0 ng/ml), respectively. A total of 60.3%, 54.6%, and 50.4% of patients had cardiovascular disease, diabetes mellitus, and heart failure, respectively. Approximately 75% of patients were with CKD stage 4–5 (6.9% stage 3a, 18.7% stage 3b, 34.2% stage 4, and 40.2% stage 5, respectively). The mean (±SD) eGFR was 21.1±13.7 ml/min per 1.73 m^2^. Patient characteristics were similar between patients with Hb <10 and ≥10 g/dl, except for the proportion of CKD stage 5 in 47.6% versus 36.3% of patients (Hb <10 versus ≥10 g/dl). Similarly, the proportion of patients with c-reactive protein >0.3 mg/dl were higher in patients with Hb <10 g/dl. Baseline characteristics summarized by iron parameters are shown in Supplemental Table 4.

**Table 1.** Baseline characteristics in the overall patient population and subgroups categorized by hemoglobin level

Characteristics	Total (*N*=26,626)	Hb <10 g/dl (*n*=9130)	Hb ≥10 g/dl (*n*=17,496)
**Age (yr)**			
Mean±SD	75.9±11.9	76.9±11.8	75.3±11.9
Sex, female, *no.* (%)	10,053 (37.8)	3903 (42.7)	6150 (35.2)
**Follow-up time (yr)**			
Mean±SD	2.5±2.3	2.1±2.2	2.7±2.3
**Hb (g/dl)**			
Mean±SD	9.9±1.2	8.7±1.3	10.6±0.3
**Ferritin (ng/ml)**			
Patients with recorded ferritin values, *no.* (%)	5688 (21.4)	2363 (25.9)	3325 (19.0)
Median (IQR)	113.2 (47.5–225.0)	121.8 (43.0–252.8)	108.0 (50.0–209.4)
**TSAT (%)**			
Patients with recorded TSAT values, *no.* (%)	945 (3.5)	331 (3.6)	614 (3.5)
Mean±SD	25.6±14.5	26.3±17.5	25.2±12.6
**CRP (mg/dl)**			
Patients with recorded CRP values, *no.* (%)	22,728 (85.4)	8064 (88.3)	14,664 (83.8)
Median (IQR)	0.6 (0.1–3.5)	0.7 (0.1–4.2)	0.5 (0.1–3.1)
CRP category, *no.* (%)^a^			
<0.3 mg/dl	9086 (40.0)	2925 (36.3)	6161 (42.0)
≥0.3 mg/dl	13,642 (60.0)	5139 (63.7)	8503 (58.0)
**eGFR value (ml/min per 1.73 m** ^**2**^ **)**			
Mean±SD	21.1±13.7	19.1±13.2	22.1±13.8
**CKD stage, *no.* (%)**			
Stage 3a	1845 (6.9)	509 (5.6)	1336 (7.6)
Stage 3b	4649 (18.7)	1380 (15.1)	3589 (20.5)
Stage 4	9112 (34.2)	2893 (31.7)	6219 (35.5)
Stage 5	10,700 (40.2)	4348 (47.6)	6352 (36.3)
**Comorbidity, *no.* (%)**			
Heart failure	13,411 (50.4)	4493 (49.2)	8918 (51.0)
Diabetes mellitus	14,545 (54.6)	4624 (50.6)	9921 (56.7)
Hypertension	19,842 (74.5)	6407 (70.2)	13,435 (76.8)
Cardiovascular disease	16,060 (60.3)	5374 (58.9)	10,686 (61.1)
Myocardial infarction	1487 (5.6)	492 (5.4)	995 (5.7)
Stroke	5218 (19.6)	1624 (17.8)	3594 (20.5)
Moderate-to-severe liver disease	345 (1.3)	132 (1.4)	213 (1.2)
Smoking history	3631 (13.6)	1139 (12.5)	2492 (14.2)
**Charlson Comorbidity Index**			
Mean±SD	3.7±2.1	3.5±2.1	3.8±2.1

Hb, hemoglobin; IQR, interquartile range; TSAT, transferrin saturation; CRP, c-reactive protein.

a The denominator is the no. of patients with available records for the corresponding laboratory values.

### Patterns of Anemia Treatment Initiation and Continuation

**Figure 2.**
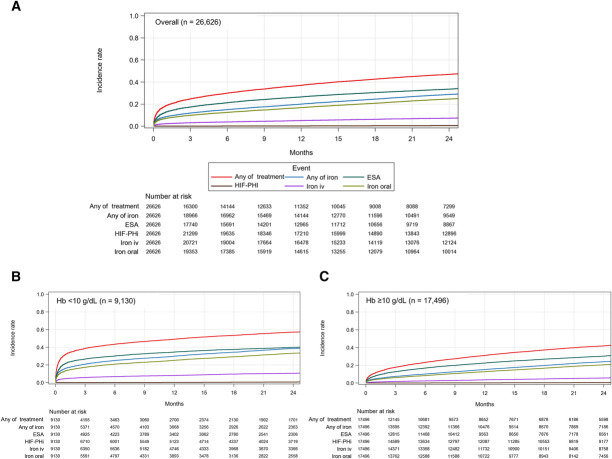
**Cumulative incidence curves of anemia treatment initiation in the overall patient population and subgroups of patients categorized by Hb level.** (A) The cumulative incidence curve in the overall patient population. The results of the subgroups of patients with (B) Hb <10 and (C) ≥10 g/dl. ESA, erythropoiesis-stimulating agent; Hb, hemoglobin; HIF-PHI, hypoxia-inducible factor prolyl hydroxylase inhibitor; iv, intravenous.

Figure 2 depicts the cumulative incidence curves of anemia treatment initiation in the overall patient population and subgroups categorized by Hb level. The cumulative incidence (95% CI) of any anemia treatment initiation within 12 months was 37.1% (36.4% to 37.7%), including ESA 26.5% (26.0% to 27.1%), iron oral 16.8% (16.4% to 17.3%), iron iv 5.1% (4.8% to 5.4%), and HIF-PHI 0.2% (0.1% to 0.3%) (Table 2). A higher incidence of treatment initiation was observed in patients with Hb <10 g/dl than in patients with Hb ≥10 g/dl, with a cumulative incidence (95% CI) of 49.1% (48.0% to 50.3%) versus 31.1% (30.3% to 31.8%) at 12 months. The initiation of iron oral and iron iv were more frequently observed in patients with TSAT <20% (27.4% for iron oral and 7.9% for iron iv at 12 months) and in patients with ferritin level <100 ng/ml (30.8% for iron oral and 11.0% for iron iv at 12 months) (Table 2). Compared with patients with stage 3 CKD, the initiation of ESA was more frequently observed in patients with stage 4–5 CKD, with cumulative incidences (95% CI) of treatment initiation at 12 months of 8.3% (7.6% to 9.0%) versus 33.0% (32.3% to 33.7%) (stage 3 versus stage 4–5) (Supplemental Figure 1).

**Table 2.** Cumulative incidence of anemia treatment initiation by treatment type in the overall patient population and patients categorized by hemoglobin level, iron parameters, and CKD stages

Treatment Type	Total	Hb <10 g/dl	Hb ≥10 g/dl	Ferritin <100 ng/ml	Ferritin ≥100 ng/ml	TSAT <20%	TSAT ≥20%	CKD Stage 3	CKD Stage 4–5
	*N*=26,626	*n*=9130	*n*=17,496	*n*=2606	*n*=3082	*n*=350	*n*=595	*n*=6814	*n*=19,812
**At 6 mo, % (95% CI)**									
Any anemia treatment	30.0 (29.4 to 30.5)	43.4 (42.3 to 44.5)	23.2 (22.6 to 23.9)	40.1 (38.2 to 42.0)	40.6 (38.8 to 42.4)	32.8 (28.0 to 38.1)	24.0 (20.6 to 27.7)	18.3 (17.3 to 19.2)	34.1 (33.4 to 34.8)
ESA	21.3 (20.7 to 21.8)	30.1 (29.0 to 31.1)	16.9 (16.3 to 17.5)	24.4 (22.8 to 26.2)	34.8 (33.0 to 36.6)	18.2 (14.5 to 22.8)	19.4 (16.4 to 22.9)	5.8 (5.3 to 6.5)	26.7 (26.1 to 27.4)
HIF-PHI	0.1 (0.1 to 0.2)	0.1 (0.1 to 0.3)	0.1 (0.1 to 0.2)	0.1 (0 to 0.4)	0.2 (0.1 to 0.5)	0 (0 to 0)	0.4 (0.1 to 1.5)	0 (0 to 0.1)	0.1 (0.1 to 0.2)
Iron oral	12.5 (12.1 to 12.9)	20.9 (20.0 to 21.8)	8.4 (8.0 to 8.9)	23.7 (22.1 to 25.4)	10.7 (9.6 to 11.9)	19.4 (15.5 to 24.1)	8.9 (6.8 to 11.7)	13.1 (12.2 to 13.9)	12.3 (11.8 to 12.8)
Iron iv	3.8 (3.6 to 4.1)	6.8 (6.3 to 7.4)	2.3 (2.2 to 2.6)	8.9 (7.9 to 10.1)	3.9 (3.2 to 4.6)	6.1 (4.0 to 9.3)	2.1 (1.2 to 3.7)	3.0 (2.6 to 3.4)	4.2 (3.9 to 4.5)
Iron oral or iv	15.0 (14.6 to 15.5)	25.0 (24.1 to 26.0)	10.1 (9.7 to 10.6)	29.1 (27.4 to 31.0)	13.3 (12.1 to 14.6)	23.9 (19.7 to 28.9)	10.3 (8.0 to 13.2)	14.4 (13.6 to 15.3)	15.3 (14.7 to 15.8)
**At 12 mo, % (95% CI)**									
Any anemia treatment	37.1 (36.4 to 37.7)	49.1 (48.0 to 50.3)	31.1 (30.3 to 31.8)	48.2 (46.2 to 50.2)	47.3 (45.4 to 49.2)	41.9 (36.7 to 47.6)	34.7 (30.8 to 38.9)	23.3 (22.3 to 24.5)	41.9 (41.1 to 42.7)
ESA	26.5 (26.0 to 27.1)	34.6 (33.5 to 35.7)	22.5 (21.8 to 23.2)	30.0 (28.2 to 31.9)	40.2 (38.3 to 42.1)	23.6 (19.3 to 28.7)	24.2 (20.8 to 28.0)	8.3 (7.6 to 9.0)	33.0 (32.3 to 33.7)
HIF-PHI	0.2 (0.1 to 0.3)	0.3 (0.2 to 0.4)	0.2 (0.1 to 0.3)	0.1 (0 to 0.4)	0.3 (0.2 to 0.7)	0 (0 to 0)	0.8 (0.3 to 2.2)	0.1 (0 to 0.2)	0.2 (0.2 to 0.3)
Iron oral	16.8 (16.4 to 17.3)	25.2 (24.2 to 26.2)	12.8 (12.2 to 13.3)	30.8 (29.0 to 32.7)	14.6 (13.3 to 16.0)	27.4 (22.7 to 32.7)	16.3 (13.3 to 19.8)	16.7 (15.8 to 17.7)	16.9 (16.3 to 17.5)
Iron iv	5.1 (4.8 to 5.4)	8.1 (7.5 to 8.8)	3.6 (3.3 to 3.9)	11.0 (9.8 to 12.3)	5.3 (4.5 to 6.2)	7.9 (5.4 to 11.5)	3.6 (2.3 to 5.6)	3.7 (3.2 to 4.2)	5.6 (5.3 to 6.0)
Iron oral or iv	20.0 (19.5 to 20.6)	29.9 (28.9 to 31.0)	15.2 (14.6 to 15.8)	37.0 (35.1 to 39.0)	18.0 (16.5 to 19.5)	32.7 (27.7 to 38.2)	18.7 (15.5 to 22.4)	18.3 (17.4 to 19.4)	20.7 (20.0 to 21.3)

Hb, hemoglobin; TSAT, transferrin saturation; CI, confidence interval; ESA, erythropoiesis-stimulating agent; HIF-PHI, hypoxia-inducible factor prolyl hydroxylase inhibitor; iv, intravenous.

**Figure 3.**
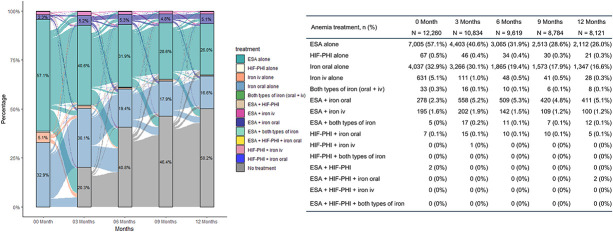
**The longitudinal patterns of anemia treatment in patients who initiated anemia treatment.** ESA, erythropoiesis stimulating agent; HIF-PHI, hypoxia-inducible factor prolyl hydroxylase inhibitor; iv, intravenous.

**Figure 4.**
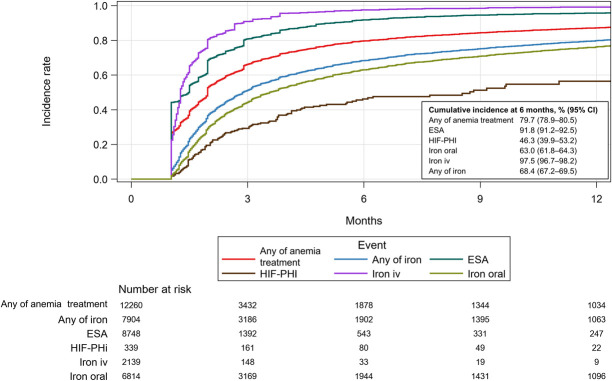
**Kaplan-Meier plot of discontinuation of anemia treatment after initiation.** Treatment discontinuation was defined as not prescribed with the same type of treatment for 30 days after the end of the last treatment episode. CI, confidence interval; ESA, erythropoiesis-stimulating agent; HIF-PHI, hypoxia-inducible factor prolyl hydroxylase inhibitor; iv, intravenous.

The longitudinal treatment patterns are shown in Figure 3. The most common treatment type at treatment initiation was ESA alone (57.1%), followed by iron oral alone (32.9%) and iron iv alone (5.1%). Iron oral was the most common treatment type in patients with ferritin <100 ng/ml (43.7%), TSAT <20% (50.2%), and patients with stage 3 CKD (55.5%) (Supplemental Figure 2). Combination therapy was rarely used at treatment initiation and increased during the follow-up, including the combination of ESA+iron oral in 5% and ESA+iron iv in 1%–2% of patients. Furthermore, 40.8% and 50.2% of patients were not on any anemia treatment at 6 and 12 months, respectively. Figure 4 shows the Kaplan-Meier plot for the discontinuation of initial treatment. The cumulative incidences of discontinuation of ESA, iron oral, iron iv, and HIF-PHI were 91.8% (91.2% to 92.5%), 63.0% (61.8% to 64.3%), 97.5% (96.7% to 98.2%), and 46.3% (39.9% to 53.2%) at 6 months after treatment initiation, respectively.

### Hb Level and Iron Parameters

The mean (±SD) Hb level and the median (IQR) ferritin level at treatment initiation were 9.3±1.5 g/dl and 93.1 ng/ml (34.6–205.5 ng/ml), respectively (Table 3). Among the anemia treatment types studied, iron iv was started at the lowest Hb level. Iron oral or iv was started at a median (IQR) ferritin level of 45.0 ng/ml (20.9–102.2 ng/ml) and a mean (±SD) TSAT of 18.7%±12.2%.

**Table 3.** Hemoglobin levels and iron parameters at anemia treatment initiation

Parameters	Any Anemia Treatment	ESA	HIF-PHI	Iron Oral	Iron iv	Iron Oral or iv
	*N*=12,260	*n*=8748	*n*=339	*n*=6814	*n*=2139	*n*=7914
**Hb (g/dl)**						
Patients with recorded Hb values, *no.* (%)^a^	12,172 (99.3)	8685 (99.3)	336 (99.1)	6759 (99.2)	2124 (99.3)	7837 (99.0)
Mean±SD	9.3±1.5	9.2±1.3	9.6±1.5	9.4±1.7	8.9±1.6	9.3±1.7
Hb category, *no.* (%)^b^						
*<10 g/dl*	8176 (67.2)	6093 (70.2)	203 (60.4)	4230 (62.6)	1560 (73.4)	5050 (64.4)
*≥10 and <12 g/dl*	3571 (29.3)	2439 (28.1)	111 (33.0)	2080 (30.8)	503 (23.7)	2333 (29.8)
*≥12 g/dl*	425 (3.5)	153 (1.8)	22 (6.5)	449 (6.6)	61 (2.9)	454 (5.8)
**Ferritin (ng/ml)**						
Patients with recorded ferritin values, *no.* (%)^a^	6671 (54.4)	4824 (55.1)	226 (66.7)	4424 (64.9)	1613 (75.4)	5208 (65.8)
Median (IQR)	93.1 (34.6–205.5)	133.0 (62.0–252.8)	130.5 (63.7–249.0)	44.0 (20.1–103.1)	47.2 (21.6–104.0)	45.0 (20.9–102.2)
Ferritin category, *no.* (%)^b^						
*<50 ng/ml*	2213 (33.2)	952 (19.7)	44 (19.5)	2399 (54.2)	841 (52.1)	2792 (53.6)
*≥50 and <100 ng/ml*	1237 (18.5)	954 (19.8)	51 (22.6)	882 (19.9)	354 (21.9)	1084 (20.8)
*≥100 ng/ml*	3221 (48.3)	2918 (60.5)	131 (58.0)	1143 (25.8)	418 (25.9)	1332 (25.6)
**TSAT (%)**						
Patients with recorded TSAT values, *no.* (%)^a^	932 (7.6)	624 (7.1)	84 (24.8)	687 (10.1)	287 (13.4)	835 (10.6)
Mean±SD	22.4±14.3	25.6±15.4	31.1±17.2	19.4±12.5	16.0±9.8	18.7±12.2
TSAT category, *no.* (%)^b^						
*<20%*	466 (50)	251 (40.2)	23 (27.4)	413 (60.1)	221 (77.0)	538 (64.4)
*≥20%*	466 (50)	373 (59.8)	61 (72.6)	274 (39.9)	66 (23.0)	297 (35.6)

ESA, erythropoiesis-stimulating agent; HIF-PHI, hypoxia-inducible factor prolyl hydroxylase inhibitor; iv, intravenous; Hb, hemoglobin; IQR, interquartile range; TSAT, transferrin saturation.

a No. of patients with corresponding laboratory values within 90 days of treatment initiation.

b The denominator is the no. of patients with available records for the corresponding laboratory values.

**Figure 5.**
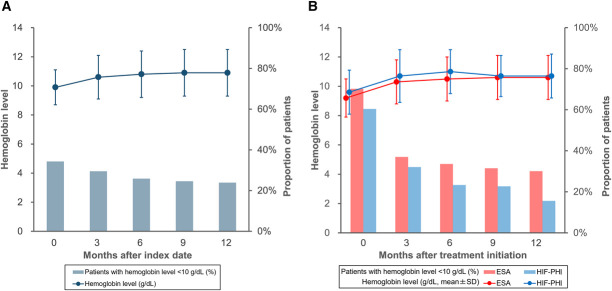
**Changes in Hb levels and the proportions of patients with Hb <10 g/dl in the overall patient population and patients treated with ESA or HIF-PHI.** (A) The Hb level in the overall patient population after the index date, and (B) the Hb level by treatment type after initiation of ESA or HIF-PHI. ESA, erythropoiesis-stimulating agent; Hb, hemoglobin; HIF-PHI, hypoxia-inducible factor prolyl hydroxylase inhibitor.

Figure 5 shows the change in Hb levels in the overall patient population and in patients treated with ESA or HIF-PHI. In the overall patient population, the mean (±SD) Hb levels at index and at 3, 6, 9, and 12 months of follow-up were 9.9±1.2, 10.6±1.5, 10.8±1.6, 10.9±1.6, and 10.9±1.6 g/dl, respectively. The proportion of patients at Hb <10 g/dl at index and at 3, 6, 9, and 12 months were 34.3%, 29.5%, 25.9%, 24.6%, and 23.9%, respectively. In patients treated with ESA or HIF-PHI (*n*=8876), the mean Hb (±SD) levels had increased from 9.3±1.3 to 10.3±1.5 g/dl at 3 months and 10.6±1.5 g/dl at 12 months after treatment initiation. In this subgroup, the proportion of patients at Hb <10 g/dl had decreased from 70.0% to 30.1% at 12 months (Supplemental Table 5).

During the follow-up period, decreasing trends in ferritin levels were observed in the overall patient population (Supplemental Figure 3). The median (IQR) ferritin levels at index and at 3, 6, 9, and 12 months in the overall patient population were 113.2 (47.5–225.0) ng/ml, 96.6 (43.0–202.2) ng/ml, 89.6 ng/dl (40.1–184.0 ng/ml), 88.0 (40.1–174.0) ng/ml, and 83.0 (39.0–165.9) ng/ml, respectively. In patients treated with ESA or HIF-PHI, the ferritin levels were decreased at 3 months but maintained at the same level thereafter. The median (IQR) ferritin levels at index and at 3, 6, 9, and 12 months were 132.2 (61.9–252.4) ng/ml, 98.0 (46.0–191.0) ng/ml, 97.9 (45.1–186.0) ng/ml, 98.5 (49.1–188.2) ng/ml, and 97.0 (47.1–182.7) ng/ml, respectively (Supplemental Table 5).

### Association between Hb Variability and the Risk of Clinical Events

**Figure 6.**
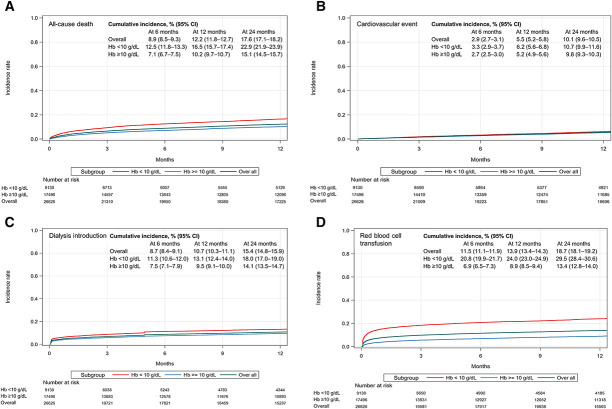
**Cumulative incidence curves of studied clinical events in the overall patient population and subgroups categorized by Hb level.** (A) The cumulative incidence curves of all-cause death. The cumulative incidence curves of (B) cardiovascular event, (C) dialysis introduction, and (D) red blood cell transfusion. CI, confidence interval; Hb, hemoglobin.

**Figure 7.**
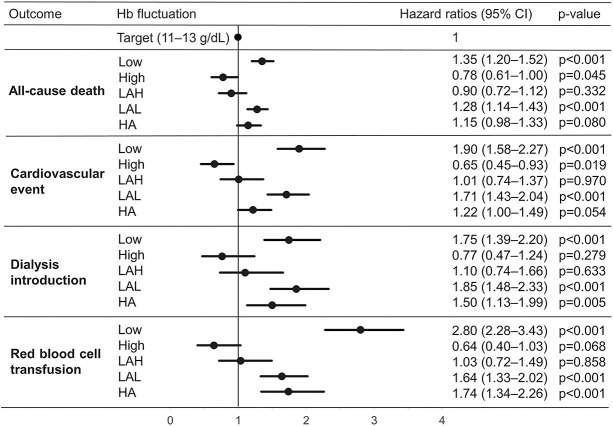
**Association between Hb fluctuation and the risk of clinical events in the time-dependent Cox proportional hazard model.** Hb fluctuation patterns were categorized into six groups: within the target Hb range (11–13 g/dl) (target), consistently below the target (low), consistently above the target (high), low amplitude fluctuation around the LAH, low-amplitude fluctuation around the LAL, and HA. Time-dependent Cox proportional hazard models were adjusted by Hb fluctuation patterns, use of erythropoiesis-stimulating agent, iron oral (including dose), iron iv, hypoxia-inducible factor prolyl hydroxylase inhibitor, red blood cell transfusion, and ferritin category (ferritin <100 or ≥100 ng/ml) as time-dependent covariates and eGFR, albumin, c-reactive protein, age, sex, cardiovascular disease, diabetes mellitus, heart failure, and etiology of kidney disease (hypertension, glomerulonephritis, renovascular disease, polycystic kidney disease, and autoimmune disease) as time-independent covariates. CI, confidential interval; HA, high-amplitude fluctuation across the target; Hb, hemoglobin; LAH, upper limit of the target; LAL, lower limit of the target.

During the follow-up period, 5991 (22.5%) deaths, 3545 (13.3%) cardiovascular events, 4231 (15.9%) dialysis introductions, and 5561 (20.9%) red blood cell transfusions were observed. The cumulative incidence curves show higher incidences of clinical events in patients with Hb <10 g/dl compared with patients with Hb ≥10 g/dl (Figure 6). In the time-dependent Cox proportional hazard models, the risks of clinical events were significantly higher in the low and LAL Hb groups than in the target Hb group (Figure 7). The hazard ratios (95% CIs) for deaths, cardiovascular events, dialysis introduction, and red blood cell transfusion in the low Hb group compared with the target Hb group were 1.35 (1.20–1.52), 1.90 (1.58–2.27), 1.75 (1.39–2.20), and 2.80 (2.28–3.43), respectively, and in the LAL Hb group were 1.28 (1.14–1.43), 1.71 (1.43–2.04), 1.85 (1.48–2.33), and 1.64 (1.33–2.02), respectively. In the HA Hb group, significantly higher risks for dialysis introduction and red blood cell transfusion were observed. These findings were consistent when the analysis was performed with a target Hb range of 11–12 g/dl (Supplemental Figure 4).

## Discussion

This large, contemporary study reports the management of anemia in a real-world cohort of >26,000 patients with NDD-CKD from multifaceted aspects, including a cross-sectional study of anemia treatment on initial Hb response and Hb fluctuations to provide comprehensive epidemiologic information on anemia management in routine clinical care.

A recent study reported that treatment initiation for any anemia treatment in a multinational cohort of patients with NDD-CKD was 40% in patients with Hb <10 g/dl, within 12 months.^15^ A similar or greater range of treatment initiation was observed in this study; nevertheless, more than half of the patients did not initiate any anemia treatment within 1 year of first identified Hb qualifying them for treatment. Consistent with a previous report from Japan,^6^ the utilization rate of ESA was low, particularly in patients with CKD stage 3. This study extends this knowledge with longitudinal information regarding treatment initiation, also allowing the time for multiple confirmations of low Hb levels. However, the ESA initiation rate remained <10% in this group, and instead, iron oral was the most prescribed anemia treatment. By contrast, the ESA initiation rate was the highest among the anemia treatment in patients with stage 4–5 CKD. These findings are suggestive of the controversies in optimal treatment algorithms for anemia management in patients with NDD-CKD. Previous clinical trials showed increased cardiovascular risks associated with the use of ESA for higher target Hb levels, introducing controversies in the treatment algorithms, such as the triggers of treatment initiation and the ideal target of Hb correction for anemia management.^10–12^ Subsequently, the Kidney Disease Improving Global Outcomes guidelines suggest that the use of ESA therapy should be individualized on the basis of the rate of the fall of Hb concentration, prior response to iron therapy, and the risk-benefit balance of ESA therapy on the basis of the patients' condition/symptoms in patients with NDD-CKD with Hb <10 g/dl.^7^ The longitudinal anemia treatment patterns and high discontinuation rates of initial treatment suggest that the anemia treatment for patients with NDD-CKD was intermittently altered rather than continuously maintained in the same regimen. Approximately half of patients were not on any anemia treatment after 12 months. Although some patients were assumed to discontinue anemia treatment after the recovery of Hb concentration to the target level, the notably high proportion of patients without any treatment suggests the need for further investigation regarding barriers for treatment continuation to maintain a stable Hb concentration in patients with NDD-CKD. The lower discontinuation rate for oral medications compared with parenteral treatments may also suggest the importance of burden related to invasive treatment as an additional aspect for treatment continuation.

Many of the patients in this study had heart failure, cardiovascular, and advanced kidney disease, which is known as cardiorenal anemia syndrome.^23^ Similarly, the risks of mortality, cardiovascular events, and dialysis introduction were even higher in the subgroup with Hb <10 g/dl. These findings underscore the importance of anemia as a treatment target to improve patients' prognoses and quality of life. We found that greater risks of mortality, cardiovascular events, dialysis introduction, and red blood cell transfusion were associated with the low and LAL Hb fluctuation groups, compared with the group with the target Hb range. HA Hb fluctuation was associated with greater risks of dialysis introduction and red blood cell transfusion and numerically but not significantly higher risks of mortality and cardiovascular events. These findings reaffirm the findings from a previous study in patients on hemodialysis, showing that increased risks of adverse clinical events associated with low, LAL, and HA fluctuations,^17^ are applicable in patients with NDD-CKD. Similarly, this study extends the finding of the increased mortality risk associated with Hb variability in patients with NDD-CKD^18^ to cardiovascular and renal events, as well as red blood cell transfusion. Transfusion reduction is an evidence-based benefit of anemia treatment. The results suggest the importance of stable Hb control to reduce the number of transfusions, which leads to the reduced risk of adverse events associated with transfusion, for example, allosensitization for future transplant. Numerous studies have shown the greater risks of cardiovascular disease and kidney disease progression associated with low Hb levels.^24–28^ Taken together, these findings underscore the importance of stable Hb control within the target Hb range. The increased risks of adverse clinical events even in the group whose Hb level was fluctuating around the lower limit of target Hb range suggest the importance of maintaining Hb level within the target range of Hb 11–13 g/dl. However, 20%–30% of patients persistently remained at Hb <10 g/dl, even in patients who initiated ESA or HIF-PHI therapy, suggesting the need for a further assessment of causality for example, ESA hyporesponse, as well as measures to correct low Hb levels in these patients. The higher c-reactive protein levels in patients with low Hb levels suggest that patients with low Hb levels and high Hb variability were more likely inflamed and sicker. These underlying conditions should be considered to affect the higher event risks in these patients. Hepcidin levels may be elevated because of the inflammatory states associated with advanced CKD. Concurrent depression of erythropoiesis by inflammation at the prevailing erythropoietin levels prevents the increase in erythroferrone release from erythroblasts that then opposes hepcidin effects on macrophages to mobilize their stored iron and increase gastrointestinal absorption of dietary iron.^29^ Together with iron deficiency, measurements of inflammatory markers and ESA response should become a part of management of resistant anemia in patients with NDD-CKD.

Evidence from biological, epidemiologic, and clinical studies that have accrued in recent years and the emergence of novel anemia therapies have resulted in the need for a re-examination of the previous recommendations for optimal anemia management in patients with CKD.^30^ For instance, studies to clarify the specific populations that are suitable for HIF-PHI therapy, such as inflamed patients and ESA hyporesponders are of interest^30,31^ while several important issues including the resolution of safety concerns need to be further clarified.^32^ In this study, iron parameters were more frequently measured in patients treated with HIF-PHIs. This finding may somehow reflect the attention of the physicians, on the basis of the recommendation issued by the Japanese Society of Nephrology and the Asian Pacific Society of Nephrology, emphasizing the importance of correcting iron deficiency before the initiation of HIF-PHIs.^33^ Iron deficiency should also be corrected before and/or during treatment with ESAs.^7^ Although, the iron therapy initiation was observed in patients with low ferritin levels, the low rate of combination therapy with iron and ESAs in this study may reflect underutilization of iron therapy compared with non-Japanese population, which may limit the generalizability of the study findings.

This study has several strengths, including the large sample size that included a broad range of patients. However, the study population was mainly comprised patients treated at hospitals, limiting the direct application of the study findings to primary care settings. Several limitations were considered. First, the study period included the period before the authorization of HIF-PHIs for clinical use (available from August 2020 for NDD-CKD). Therefore, the number of patients prescribed HIF-PHIs was small during the study period. Although the possibility of underreporting the treatment initiation rate and the selection of patients for new therapies may not be fully denied, the early insight regarding the Hb and iron parameters and the longitudinal treatment patterns may provide useful information. Similarly, the iron parameters were not measured in all patients. Therefore, these results may not represent the overall study population. Second, RWD data collected only structured information. Hence, qualitative information such as reasons for treatment initiation/discontinuation and persistently low Hb levels cannot be determined. Finally, the association found in the observational study cannot be directly considered as a causal relationship. The analysis for the association between Hb fluctuation patterns and the risks of clinical events included both patients on treatment and not on treatment. Therefore, the results do not suggest or recommend a target Hb range for clinical use. It should also be noted that there are no randomized controlled trials with any form of anemia treatment to support the reduction of adverse events by more consistent anemia treatment. Whether consistent anemia treatment results in fewer adverse events remains to be explored in properly designed clinical trials.

In this contemporary study, we have reported comprehensive epidemiologic information on anemia management in patients with NDD-CKD from routine clinical care. Despite the increased burden on the patients' quality of life and the mortality and morbidity of anemia, the treatment initiation rate was low, and for patients treated, 20%–30% persistently remained at low Hb levels. The findings underscore the importance of maintaining a stable Hb concentration within the target range to reduce the risk of mortality and morbidity in patients with NDD-CKD while highlighting the suboptimal and heterogeneous treatment of anemia in clinical practice. The findings from this study should provide further clarity in optimizing algorithms for anemia management in patients with NDD-CKD for a further improvement in patient outcomes.

## Disclosures

This study and the corresponding analyses were supported and funded by Bayer. All authors from Bayer participated in the organization of the study design, interpretation of the results, contribution to the manuscript drafts and revisions, and the decision to approve publication of the final manuscript. T. Hayasaki, Y. Ishida, G. James, S. Okami, and K. Yoshikawa-Ryan, are employed by Bayer. G. James reports ownership interest in Bayer Stock. T. Kuragano reports the following: Employer: Division of Kidney and Dialysis, Department of Internal Medicine, Hyogo Medical University; Research Funding: T. Kuragano has research grants from Kissei Pharmaceutical Co., Ltd., Ono Pharmaceutical Co., Ltd.; and Speakers Bureau: Kuragano has speaker bureau from Astellas Pharma Inc., AstraZeneca Plc., Bayer Yakuhin, Ltd., Fuso Pharmaceutical Industries, Ltd., Kyowa Kirin, and Mitsubishi Tanabe Pharma Corporation, and consultancy for Astellas Pharma Inc., Bayer Yakuhin, Ltd., and Mitsubishi Tanabe Pharma Corporation. T. Kimura and H. Uenaka are employed by Real World Data Co. Ltd., which supports the maintenance of the RWD database owned by the Health, Clinic and Education Information Evaluation Institute (Kyoto, Japan). In this study, Real World Data Co. Ltd. performed the statistical analysis in accordance with the statistical analysis plan. S. Tanaka-Mizuno is affiliated with the joint research chair at Kyoto University funded by Real World Data Co. Ltd., and has received consulting fees for the biostatistics from Real World Data Co. Ltd.

## Supplementary Material

## Data Availability

Partial restrictions to the data and/or materials apply. Each individual data sharing request will be reviewed based on the sponsor's data sharing policy.

## Supplemental Material

Method details.

Time-dependent Cox proportional models to assess the risks of adverse clinical events associated with hemoglobin fluctuation.

Reporting checklist.

Supplemental tables and figures.

Supplemental Table 1. List of comorbidities.

Supplemental Table 2. Definitions of adverse clinical events collected in this study.

Supplemental Table 3. STROBE checklist.

Supplemental Table 4. Baseline characteristics in subgroups categorized by iron parameters.

Supplemental Table 5. Hemoglobin and ferritin levels by treatment type during the 12 months after treatment initiation.

Supplemental Figure 1. Cumulative incidence curves of anemia treatment initiation in subgroups categorized by TSAT, ferritin, or CKD stages.

Supplemental Figure 2. Longitudinal anemia treatment patterns in patients categorized by hemoglobin level, ferritin, TSAT, or CKD stages.

Supplemental Figure 3. Changes in ferritin levels in the overall patient population during the follow-up period.

Supplemental Figure 4. Association between hemoglobin fluctuation and the risk of clinical events in the time-dependent Cox proportional hazard models under the condition of a target hemoglobin range of 11–12 g/dl.

References
