# Supplementary material for: Anemia Treatment, Hemoglobin Variability, and Clinical Events in Patients With Nondialysis-Dependent CKD in Japan
Source: Kidney360. 2023 Jul 10;4(9):e1223–35. doi: 10.34067/KID.0000000000000204 (PMC10547228; doi:10.34067/KID.0000000000000204)
Supplement: Supplementary file 1 [file kidney360-4-e1223-s001.pdf]

APPENDIX

Table of contents 1
Method details 2
Time-dependent Cox proportional models to assess the risks of adverse clinical events associated with hemoglobin fluctuation 2
Reporting checklist 3
Supplementary tables and figures 4
Table S1. List of comorbidities 4
Table S2. Definitions of adverse clinical events collected in the study. 5
Table S3. STROBE Checklist 6
Table S4. Baseline characteristics in subgroups categorized by iron parameters 8
Table S5. Hemoglobin and ferritin levels by treatment type during the 12 months after treatment initiation 9
Figure S1. Cumulative incidence curves of anemia treatment initiation in subgroups categorized by TSAT, ferritin, or CKD stages 11
Figure S2. Longitudinal anemia treatment patterns in patients categorized by hemoglobin level, ferritin, TSAT, or CKD stages. 12
Figure S3. Changes in ferritin levels in the overall patient population during the follow up period. 14
Figure S4 Association between hemoglobin fluctuation and the risk of clinical events in the time-dependent Cox proportional hazard models under the condition of a target hemoglobin range of 11–12 g/dL. 15
References 16

Method details

Time-dependent Cox proportional models to assess the risks of adverse clinical events associated with hemoglobin fluctuation

The risks of adverse clinical events can be affected not only by the baseline characteristics of patients, but also by parameters continuously changing over the follow up period. Therefore, the classical Cox hazard model may not be suitable for assessing the risks of clinical events associated with transitioning hemoglobin fluctuation. We applied the Anderson and Gill model[1] for the analysis to evaluate the risk of transitioning hemoglobin (Hb) fluctuation patterns associated with the risk of clinical events. This model treats the observational period of each patient as an independent factor. The observational periods of each patient were divided into every 6-month period in which the time-dependent covariates were collected. In addition, the occurrence of clinical events was also assessed in each 6-month period. The time-dependent covariates were modeled to evaluate the risk associations between the time-dependent covariates and the clinical events observed in the period following the assessment period in which the time-dependent covariates were collected (Fig.). Time-independent covariates were collected for the baseline period, based on the information collected for 6 months prior to the index date. The analysis was performed in the overall patient population from the index date to the end of the follow-up period.

Day-181, Day -1 [baseline period]	Day0, Day 180	Day181, Day360	Day361, Day540	Day541, 720	--- the end of follow-up period
Time-independent covariates					
Time-dependent covariates assessment period 1	Event assessment period 1				
	Time-dependent covariates assessment period 2	Event assessment period 2			
		Time-dependent covariates assessment period 3	Event assessment period 3		
			Time-dependent covariates assessment period 4	Event assessment period 4	

Fig. Assessment windows in the time-dependent Cox proportional hazard models

The models were adjusted by Hb fluctuation patterns, use of an erythropoiesis stimulating agent, iron oral (including dose), iron intravenous, hypoxia-inducible factor prolyl hydroxylase domain enzyme inhibitor, red-blood-cell transfusion, and ferritin category (ferritin <100 ng/mL or ≥100 ng/mL) as time-dependent covariates, and estimated glomerular filtration rate, albumin, c-reactive protein, age, sex, cardiovascular disease, diabetes mellitus, heart failure, and etiology of kidney disease (hypertension, glomerulonephritis, renovascular disease, polycystic kidney disease, and auto-immune disease) as time-independent covariates. The Hb fluctuation patterns were categorized into six groups

based on the previous literature[2,3]: within the target Hb range (target); consistently below the target (Low); consistently above the target (high); low-amplitude fluctuation around the upper limit of the target (LAH); low-amplitude fluctuation around the lower limit of the target (LAL); and, high amplitude fluctuation across the target range (HA). The target Hb range was set as Hb of 11–13 g/dL, based on the Japanese guidelines and the previous study.[3-5] The sensitivity analysis was performed using the target Hb range of 11–12 g/dL.

The hazard ratios of clinical events for each Hb fluctuation group compared to target Hb group were calculated with point estimates and 95% confidence intervals.

Reporting checklist

This article was written following the STROBE (STrengthening the Reporting of OBservational studies in Epidemiology) statement,[6] details of which are further elaborated in Table S3.

Supplementary tables and figures

Table S1. List of comorbidities

Comorbidity	Definition (ICD-10 code)
Major bleeding	Intracranial: I60, I61, I62, I63.8, I69.0, I69.1<br>Retroperitoneal: K65.9 (5672027), K66.1<br>Pericardial: I23.0, I31.2, I31.2, I21.3 (8847041)<br>Intramuscular: S39.0 (8846320), S86.9 (8850129), S46.9 (8850233), S56.8 (8850254), S76.4 (8850269), S76.1 (8850271), S76.3 (8850272), S86.1 (8850322), T14.6 (9249009)<br>Intramuscular with compartment syndrome: M62.26, M62.29, T79.6 (8833907)
Malignant tumor	C00–C79, C80–C90, C96
Hypertension	D350 (8844144), I10, I11, I12, I13, I15
Diabetes mellitus	E10–E14, R73.0
Cardiovascular disease	Myocardial infarction: I21, I22<br>Unstable angina pectoris: I20.0<br>Heart failure: E05.9 (2429015), I09.9, I11.0, I50<br>Stroke: I60, I61, I63
Myocardial infarction	I21, I22
Heart failure	E05.9 (2429015), I09.9, I11.0, I50
Stroke	I60, I61, I63
Moderate-to-severe liver disease	I85.0, I85.9, I86.4, I98.2, K70.4, K71.1, K72.1, K72.9, K76.5, K76.6, K76.7
CKD	N18, N19
CKD stage 3a	A combination of ICD-10 codes and baseline eGFR value from 45–59 $mL/min/1.73m^2$
CKD stage 3b	A combination of ICD-10 codes and baseline eGFR value from 30–44 $mL/min/1.73m^2$
CKD stage 4	A combination of ICD-10 codes and baseline eGFR value from 15–29 $mL/min/1.73m^2$
CKD stage 5	A combination of ICD-10 codes and baseline eGFR value <15 $mL/min/1.73m^2$
Etiology	
Glomerulonephritis	N00, N01, N02, N03, N04, N05, N06, N07
Renovascular disease	I15.0, Q27.1, I70.1
Polycystic kidney disease	Q61
Auto-immune disease	D86, D89.1, K50, L40.0, M05, M06.8, M06.9, M30.0, M30.1, M31.0, M31.6, M32.1, M32.8, M32.9, M34.0, M34.1, M34.2, M34.8, M34.9, M35.0, M35.1, I77.6

CKD, chronic kidney disease; ICD-10, international classification of disease, 10th revision; eGFR, estimated glomerular filtration rate

Table S2. Definitions of adverse clinical events collected in the study.

Clinical outcomes	Definitions	Observed number of events in the overall patient population, n (%)
All-cause death	All death information and death record extracted from the electronical medical records in the follow-up period	5,991 (22.5)
Cardiovascular event	Hospitalizations co-occurred with disease records of myocardial infarction (ICD-10: I21, I22), unstable angina pectoris (ICD-10: I20.0), stroke (I60, I61, I63), or heart failure (ICD-10: I09.9, I11.0, I50, or E05.9 (2409015))	3,545 (13.3)
Dialysis introduction	Records of dialysis procedure codes more than three times for consecutive treatment: J038 (140007710, 140036710, 140051010, 140051110, 140052810, 140007810, 140036810, 140037910, 140038010, 140057810, 140057910, 140058010, 140058110, 140058210, 140058310, 140058410, 140058510, 140058610, 140059310, 140059410, 140059510, 140060210, 140060310, 140060410, 140060510, 140060610, 140060710, 140060810, 140060910, 140061010), J038-2 (140029850), J42 (140008510, 140008810)	4,231 (15.9)
Red-blood-cell transfusion	Procedure codes of red-blood-cell transfusion: K920 (150224910, 150286310)	5,561 (20.9)

Table S3. STROBE Checklist

	Item No	Recommendation	Page No
**Title and abstract**	1	(*a*) Indicate the study's design with a commonly used term in the title or the abstract	1
		(*b*) Provide in the abstract an informative and balanced summary of what was done and what was found	2
**Introduction**			
Background/rationale	2	Explain the scientific background and rationale for the investigation being reported	5
Objectives	3	State specific objectives, including any prespecified hypotheses	5
**Methods**			
Study design	4	Present key elements of study design early in the paper	6
Setting	5	Describe the setting, locations, and relevant dates, including periods of recruitment, exposure, follow-up, and data collection	6
Participants	6	(*a*) *Cohort study*—Give the eligibility criteria, and the sources and methods of selection of participants. Describe methods of follow-up	6
		(*b*) For matched studies, give matching criteria and number of exposed and unexposed	N/A
Variables	7	Clearly define all outcomes, exposures, predictors, potential confounders, and effect modifiers. Give diagnostic criteria, if applicable	6
Data sources/ measurement	8	For each variable of interest, give sources of data and details of methods of assessment (measurement). Describe comparability of assessment methods if there is more than one group	6
Bias	9	Describe any efforts to address potential sources of bias	12
Study size	10	Explain how the study size was arrived at	8
Quantitative variables	11	Explain how quantitative variables were handled in the analyses. If applicable, describe which groupings were chosen and why	7
Statistical methods	12	(*a*) Describe all statistical methods, including those used to control for confounding	7
		(*b*) Describe any methods used to examine subgroups and interactions	7
		(*c*) Explain how missing data were addressed	7
		(*d*) If applicable, explain how loss to follow-up was addressed	N/A
		(*e*) Describe any sensitivity analyses	7

Continued on next page

**Results**			
Participants	13	(a) Report numbers of individuals at each stage of study—e.g., numbers potentially eligible, examined for eligibility, confirmed eligible, included in the study, completing follow-up, and analysed	8
		(b) Give reasons for non-participation at each stage	8
		(c) Consider use of a flow diagram	8
Descriptive data	14	(a) Give characteristics of study participants (e.g., demographic, clinical, social) and information on exposures and potential confounders	8
		(b) Indicate number of participants with missing data for each variable of interest	23
		(c) Summarise follow-up time (e.g., average and total amount)	8
Outcome data	15	Report numbers of outcome events or summary measures over time	10
Main results	16	(*a*) Give unadjusted estimates and, if applicable, confounder-adjusted estimates and their precision (e.g., 95% confidence interval). Make clear which confounders were adjusted for and why they were included	10
		(*b*) Report category boundaries when continuous variables were categorized	7
		(*c*) If relevant, consider translating estimates of relative risk into absolute risk for a meaningful time period	N/A
Other analyses	17	Report other analyses done—e.g., analyses of subgroups and interactions, and sensitivity analyses	10
**Discussion**			
Key results	18	Summarise key results with reference to study objectives	10
Limitations	19	Discuss limitations of the study, taking into account sources of potential bias or imprecision. Discuss both direction and magnitude of any potential bias	12
Interpretation	20	Give a cautious overall interpretation of results considering objectives, limitations, multiplicity of analyses, results from similar studies, and other relevant evidence	12
Generalisability	21	Discuss the generalisability (external validity) of the study results	12
**Other information**			
Funding	22	Give the source of funding and the role of the funders for the present study and, if applicable, for the original study on which the present article is based	1, 12

Table S4. Baseline characteristics in subgroups categorized by iron parameters.

	Ferritin <100 ng/mL (N = 2,606)	Ferritin ≥100 ng/mL (N = 3,082)	TSAT <20 % (N = 350)	TSAT ≥20 % (N = 595)
Age (years)				
mean±SD	73.3±12.9	74.1±12.3	72.7±13.3	72.3±12.5
Gender, female, n (%)	1,134 (43.5)	984 (31.9)	152 (43.4)	243 (40.8)
Follow up time (years)				
mean±SD	3.2±2.5	2.6±2.3	3.1±2.6	3.2±2.6
Hb (g/dL)				
mean±SD	9.7±1.4	9.6±1.4	9.8±1.3	9.9±1.2
Ferritin (ng/mL)				
Patients with recorded ferritin values, n (%)	2,606 (100)	3,082 (100)	312 (89.1)	521 (87.6)
median (IQR)	43.0 (22.7–69.7)	211.4 (147.0–348.0)	53.4 (24.3–116.2)	122.0 (56.9–217.2)
TSAT (%)				
Patients with recorded TSAT values, n (%)	445 (17.1)	388 (12.6)	350 (100)	595 (100)
mean±SD	21.6±11.4	29.6±15.4	13.1±4.5	32.9±13.3
eGFR value (mL/min/1.73m^2)				
mean±SD	17.8±14.1	17.8±12.9	15.2±14.4	15.2±14.2
CKD stage, n (%)				
Stage 3a	164 (6.3)	141 (4.6)	23 (6.6)	34 (5.7)
Stage 3b	361 (13.9)	436 (14.1)	37 (10.6)	62 (10.4)
Stage 4	706 (27.1)	900 (29.2)	62 (17.7)	122 (20.5)
Stage 5	1,375 (52.8)	1,605 (52.1)	228 (65.1)	377 (63.4)
Comorbidity, n (%)				
Heart failure	1,406 (54.0)	1,595 (51.8)	187 (53.4)	295 (49.6)
Diabetes mellitus	1,436 (55.1)	1,668 (54.1)	209 (59.7)	328 (55.1)
Hypertension	2,020 (77.5)	2,296 (74.5)	264 (75.4)	447 (75.1)
Cardiovascular disease	1,650 (63.3)	1,871 (60.7)	240 (68.6)	364 (61.2)
Myocardial infarction	139 (5.3)	149 (4.8)	40 (11.4)	48 (8.1)
Stroke	557 (21.4)	558 (18.1)	93 (26.6)	119 (20.0)
Moderate to severe liver disease	29 (1.1)	40 (1.3)	5 (1.4)	16 (2.7)
Smoking history	231 (8.9)	439 (14.2)	23 (6.6)	44 (7.4)
Charlson comorbidity index				
mean±SD	3.7±2.1	3.7±2.1	4.0±2.3	3.7±2.4

SD, standard deviation; Hb, hemoglobin; IQR, inter quartile range; TSAT, transferrin saturation; CKD, chronic kidney disease; eGFR, estimated glomerular filtration rate.

Table S5. Hemoglobin and ferritin levels by treatment type during the 12 months after treatment initiation.

		ESA or HIF-PHI N = 8,876	ESA N = 8,748	HIF-PHI N = 339
Hb level (g/dL)				
0 months	N	8,812	8,685	336
	mean±SD	9.3±1.3	9.2±1.3	9.6±1.5
	Hb category, n (%)*			
	<10 g/dL	6,169 (70.0)	6,093 (70.2)	203 (60.4)
	≥10 and <12 g/dL	2,482 (28.2)	2,439 (28.1)	111 (33.0)
	≥12 g/dL	161 (1.8)	153 (1.8)	22 (6.5)
3 months	N	6,377	6,296	221
	mean±SD	10.3±1.5	10.3±1.5	10.7±1.8
	Hb category, n (%)*			
	<10 g/dL	2,362 (37.0)	2,332 (37.0)	71 (32.1)
	≥10 and <12 g/dL	3,201 (50.2)	3,167 (50.3)	94 (42.5)
	≥12 g/dL	814 (12.8)	797 (12.7)	56 (25.3)
6 months	N	5,333	5,276	159
	mean±SD	10.5±1.5	10.5±1.5	11.0±1.5
	Hb category, n (%)*			
	<10 g/dL	1,786 (33.5)	1,772 (33.6)	37 (23.3)
	≥10 and <12 g/dL	2,758 (51.7)	2,726 (51.7)	86 (54.1)
	≥12 g/dL	789 (14.8)	778 (14.7)	36 (22.6)
9 months	N	4,653	4,615	97
	mean±SD	10.6±1.5	10.6±1.5	10.7±1.4
	Hb category, n (%)*			
	<10 g/dL	1,460 (31.4)	1,452 (31.5)	22 (22.7)
	≥10 and <12 g/dL	2,417 (51.9)	2,394 (51.9)	61 (62.9)
	≥12 g/dL	776 (16.7)	769 (16.7)	14 (14.4)
12 months	N	4,128	4,116	45
	mean±SD	10.6±1.5	10.6±1.5	10.7±1.5
	Hb category, n (%)*			
	<10 g/dL	1,241 (30.1)	1,239 (30.1)	7 (15.6)
	≥10 and <12 g/dL	2,137 (51.8)	2,128 (51.7)	33 (73.3)
	≥12 g/dL	750 (18.2)	749 (18.2)	5(11.1)
Ferritin level (ng/mL)				
0 months	N	4,906	4,824	226
	median (IQR)	132.2 (61.9–252.4)	133.0 (62.0–252.8)	130.5 (63.7–249.0)
	Ferritin category, n (%)*			
	<50 ng/mL	977 (19.9)	952 (19.7)	44 (19.5)
	≥50 and <100 ng/mL	971 (19.8)	954 (19.8)	51 (22.6)
	≥100 ng/mL	2,958 (60.3)	2,918 (60.5)	131 (58.0)
3 months	N	2,606	2,556	139
	median (IQR)	98.0 (46.0–191.0)	98.0 (46.0–189.5)	121.1 (56.0–262.0)
	Ferritin category, n (%)*			
	<50 ng/mL	709 (27.2)	694 (27.2)	30 (21.6)
	≥50 and <100 ng/mL	622 (23.9)	611 (23.9)	32 (23.0)
	≥100 ng/mL	1,275 (48.9)	1,251 (48.9)	77 (55.4)
6 months	N	2,201	2,160	102
	median (IQR)	97.9 (45.1–186.0)	97.0 (45.0–185.4)	149.3 (81.9–217.3)
	Ferritin category, n (%)*			

	<50 ng/mL	606 (27.5)	598 (27.7)	17 (16.7)
	≥50 and <100 ng/mL	510 (23.2)	502 (23.2)	21 (20.6)
	≥100 ng/mL	1,085 (49.3)	1,060 (49.1)	64 (62.7)
9 months	N	1,891	1,864	67
	median (IQR)	98.5 (49.1–188.2)	98.0 (48.9–188.0)	140.6 (78.1–244.0)
	Ferritin category, n (%)*			
	<50 ng/mL	475 (25.1)	472 (25.3)	8 (11.9)
	≥50 and <100 ng/mL	483 (25.5)	477 (25.6)	13 (19.4)
	≥100 ng/mL	933 (49.3)	915 (49.1)	46 (68.7)
12 months	N	1,737	1,728	29
	median (IQR)	97.0 (47.1–182.7)	96.9 (47.0–182.6)	172.1 (110.0–264.0)
	Ferritin category, n (%)*			
	<50 ng/mL	453 (26.1)	452 (26.2)	1 (3.4)
	≥50 and <100 ng/mL	439 (25.3)	438 (25.3)	6 (20.7)
	≥100 ng/mL	845 (48.6)	838 (48.5)	22 (75.9)

SD, standard deviation; IQR, interquartile range; Hb, hemoglobin; ESA, erythropoiesis stimulating agent; HIF-PHI, hypoxia-inducible factor prolyl hydroxylase domain enzyme inhibitor.

*The denominator is the number of patients with available records for the corresponding laboratory values.

Figure S1. Cumulative incidence curves of anemia treatment initiation in subgroups categorized by TSAT, ferritin, or CKD stages.

Panels (A) and (B) show the cumulative incidence curves of anemia treatment initiation in the subgroups of patients with ferritin <100 ng/mL and ≥100 ng/mL, respectively. Panels (C) and (D) show the cumulative incidence curves in patients with TSAT <20% and ≥20%, respectively. Panels (E) and (F) show the cumulative incidence curves in patients with CKD stage 3 and stage 4–5, respectively. TSAT, transferrin saturation; CKD, chronic kidney disease; ESA, erythropoietin stimulating agent; HIF-PHI, hypoxia-inducible factor prolyl hydroxylase domain enzyme inhibitor.

(A) Ferritin <100 ng/mL (n = 2,606)

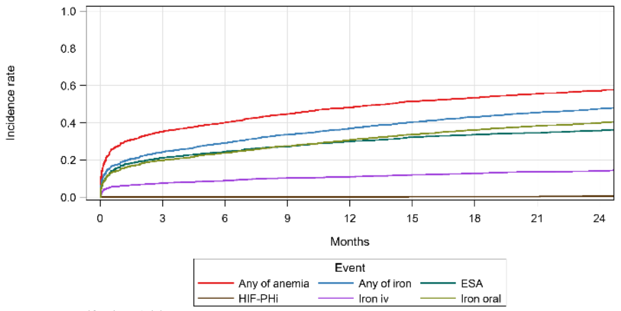

Number at risk

	0	3	6	9	12	15	18	21	24
Any of anemia	2606	1525	1358	1194	1076	959	865	784	724
Any of iron	2606	1770	1583	1411	1287	1152	1033	942	866
ESA	2606	1823	1661	1521	1405	1292	1187	1110	1037
HIF-PHi	2606	2287	2163	2049	1961	1849	1730	1636	1547
Iron iv	2606	2126	1986	1859	1769	1650	1534	1445	1356
Iron oral	2606	1868	1696	1530	1401	1270	1153	1056	983

(B) Ferritin ≥100 ng/mL (n = 3,082)

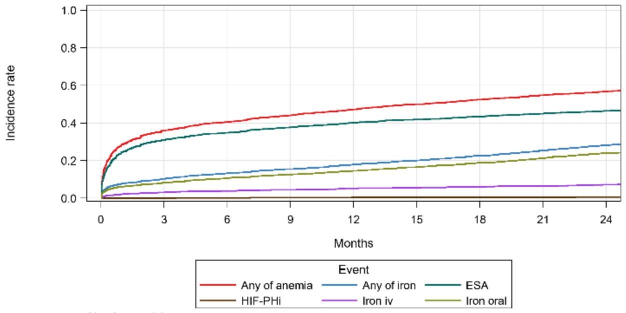

Number at risk

	0	3	6	9	12	15	18	21	24
Any of anemia	3082	1633	1411	1273	1135	1008	903	814	737
Any of iron	3082	2272	2026	1872	1706	1555	1404	1267	1154
ESA	3082	1741	1530	1399	1266	1146	1050	962	889
HIF-PHi	3082	2506	2300	2172	2038	1897	1774	1654	1559
Iron iv	3082	2444	2231	2095	1952	1815	1687	1565	1469
Iron oral	3082	2318	2077	1928	1767	1613	1463	1327	1213

(C) TSAT <20% (n = 350)

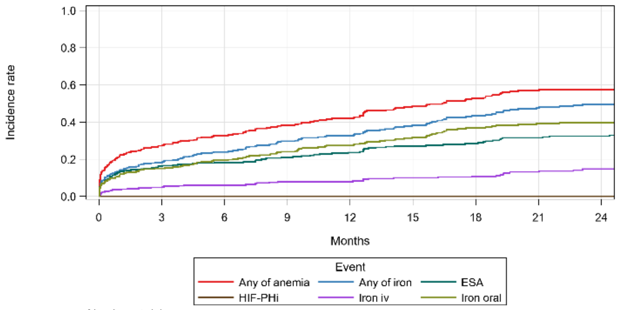

Number at risk

	0	3	6	9	12	15	18	21	24
Any of anemia	350	228	197	175	155	127	112	96	89
Any of iron	350	251	215	192	174	146	128	111	100
ESA	350	261	237	221	200	176	166	148	138
HIF-PHi	350	303	280	269	252	233	222	205	194
Iron iv	350	290	264	248	232	210	199	177	163
Iron oral	350	261	228	208	189	162	144	131	123

(D) TSAT ≥20% (n = 595)

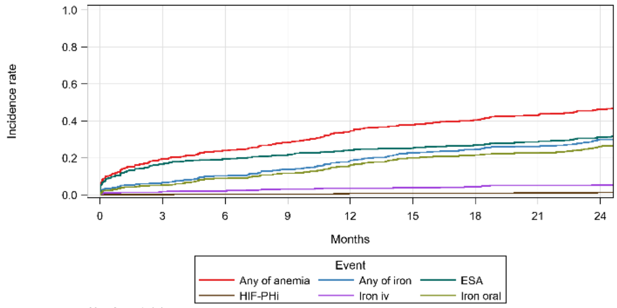

Number at risk

	0	3	6	9	12	15	18	21	24
Any of anemia	595	428	385	348	308	281	255	233	208
Any of iron	595	495	442	403	367	332	301	280	252
ESA	595	441	408	381	353	332	307	286	263
HIF-PHi	595	529	490	466	444	421	391	369	351
Iron iv	595	521	481	453	432	409	378	354	338
Iron oral	595	502	449	413	377	342	311	292	264

(E) CKD stage 3 (n = 6,814)

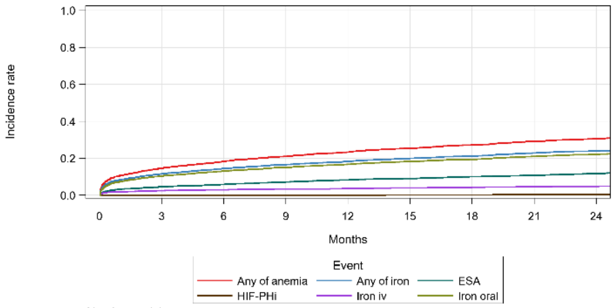

Number at risk

	0	3	6	9	12	15	18	21	24
Any of anemia	6814	4837	4315	3916	3606	3261	2950	2672	2427
Any of iron	6814	5008	4510	4139	3836	3495	3186	2906	2651
ESA	6814	5386	4924	4541	4245	3918	3589	3293	3021
HIF-PHi	6814	5634	5216	4883	4615	4290	3970	3681	3409
Iron iv	6814	5510	5087	4747	4475	4150	3830	3554	3203
Iron oral	6814	5062	4566	4194	3894	3548	3242	2952	2692

(F) CKD stage 4–5 (n = 19,812)

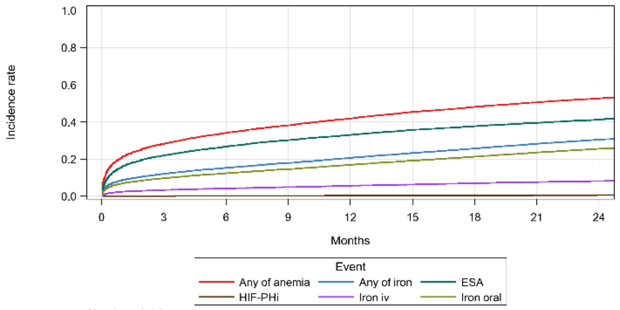

Number at risk

	0	3	6	9	12	15	18	21	24
Any of anemia	19812	11463	9829	8717	7746	6784	6058	5416	4872
Any of iron	19812	13958	12452	11330	10308	9275	8410	7585	6898
ESA	19812	12354	10767	9660	8720	7794	7067	6426	5846
HIF-PHi	19812	15665	14419	13463	12595	11709	10920	10162	9487
Iron iv	19812	15211	13917	12917	12003	11083	10289	9522	8841
Iron oral	19812	14291	12819	11725	10721	9707	8837	8012	7322

Figure S2. Longitudinal anemia treatment patterns in patients categorized by hemoglobin level, ferritin, TSAT, or CKD stages.

(A) Hb <10 g/dL (n = 4,710)

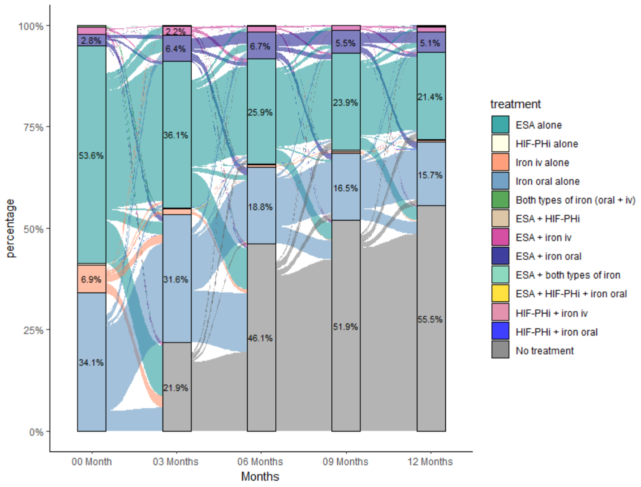

(B) Hb ≥10 g/dL (n = 7,550)

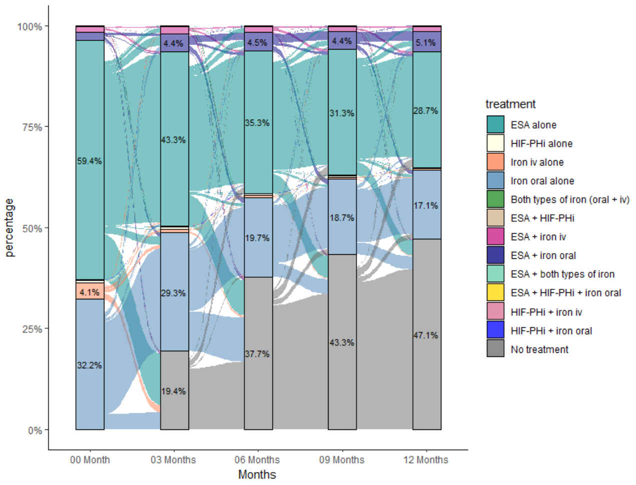

(C) Ferritin <100 ng/mL (n = 1,647)

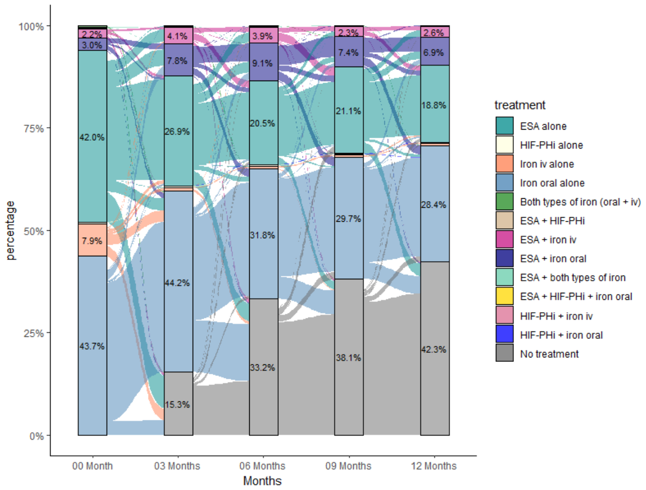

(D) Ferritin ≥100 ng/mL (n = 1,696)

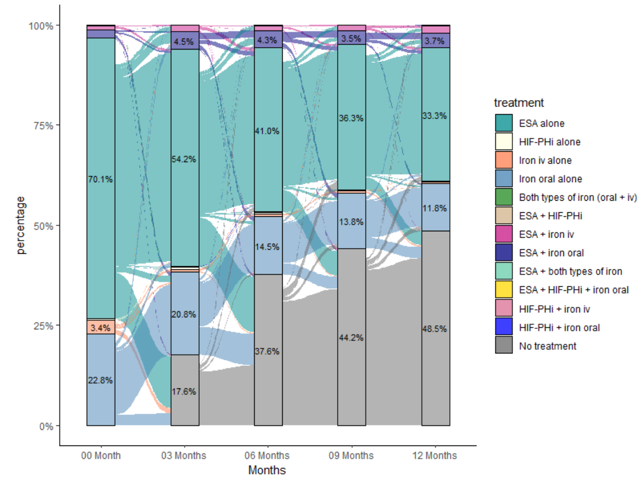

(E) TSAT <20% (n = 225)

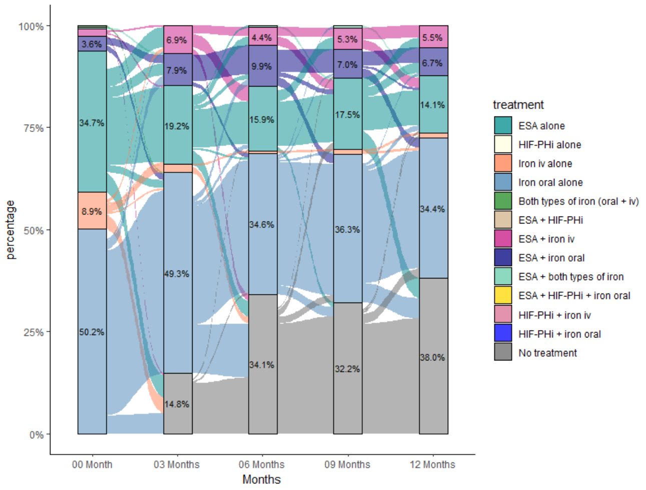

(F) TSAT ≥20% (n = 336)

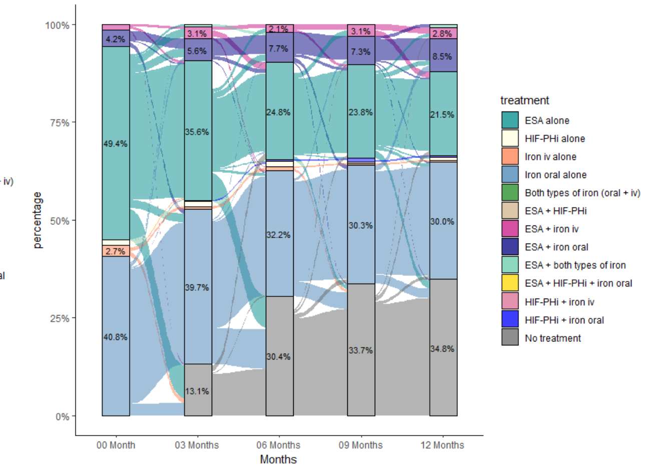

(G) CKD stage 3 (n = 2,195)

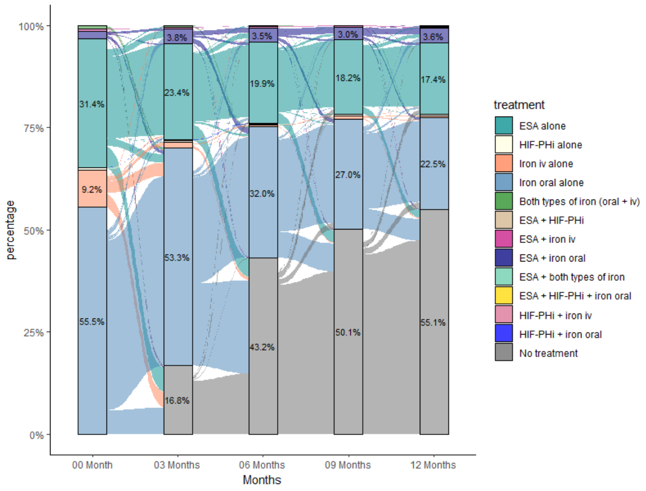

(H) CKD stage 4–5 (n = 10,065)

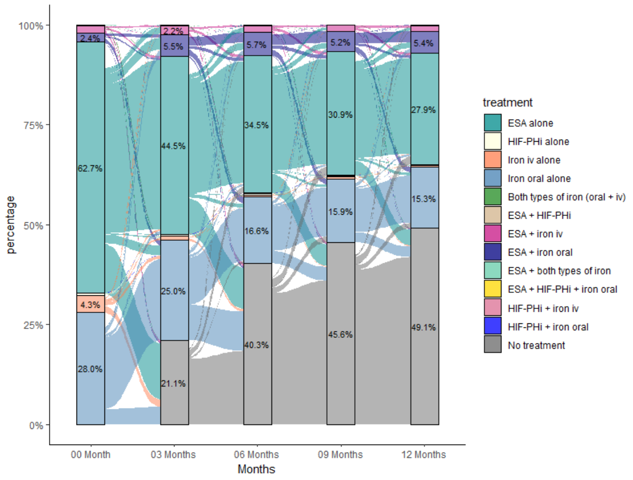

Panels (A) and (B) show the longitudinal anemia treatment patterns in the subgroups of patients with Hb <10 g/dL and ≥10 g/dL, respectively. Panels (C) and (D) show the longitudinal anemia treatment patterns in patients with ferritin <100 ng/mL and ≥100 ng/mL, respectively. Panels (E) and (F) show the longitudinal anemia treatment patterns in patients with TSAT <20% and ≥20%, respectively. Panels (G) and (H) show the results in patients with CKD stage 3 and stage 4–5, respectively. Hb, hemoglobin; TSAT, transferrin saturation; CKD, chronic kidney disease; ESA, erythropoiesis stimulating agent; HIF-PHI, hypoxia-inducible factor prolyl hydroxylase domain enzyme inhibitor.

Figure S3. Changes in ferritin levels in the overall patient population during the follow up period.

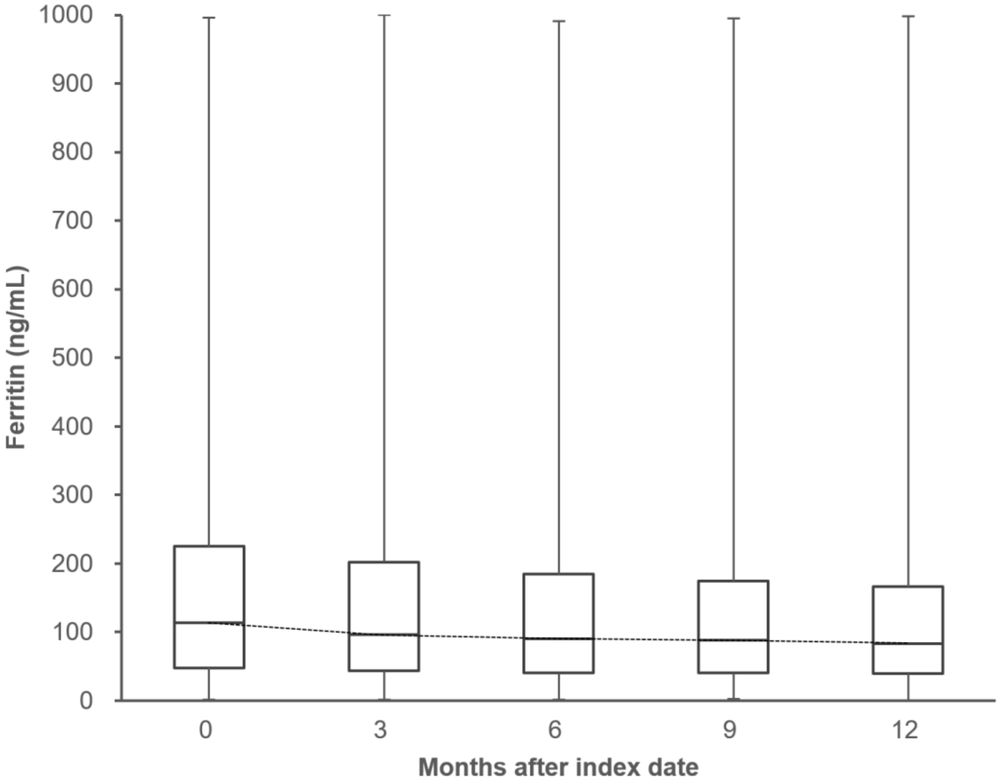

Figure S4 Association between hemoglobin fluctuation and the risk of clinical events in the time-dependent Cox proportional hazard models under the condition of a target hemoglobin range of 11–12 g/dL.

Hb fluctuation patterns were categorized into six groups: within the target Hb range (11–13 g/dL) (target); consistently below the target (low); consistently above the target (high); low-amplitude fluctuation around the upper limit of the target (LAH); low-amplitude fluctuation around the lower limit of the target (LAL); and, high amplitude fluctuation across the target (HA). Time-dependent Cox proportional hazard models were adjusted by Hb fluctuation patterns, use of an erythropoiesis stimulating agent, iron oral (including dose), iron iv, hypoxia-inducible factor prolyl hydroxylase domain enzyme inhibitor, red-blood-cell transfusion, and ferritin category (ferritin <100 ng/mL or ≥100 ng/mL) as time-dependent covariates, and estimated glomerular filtration rate, albumin, c-reactive protein, age, sex, cardiovascular disease, diabetes mellitus, heart failure, and etiology of kidney disease (hypertension, glomerulonephritis, renovascular disease, polycystic kidney disease, and auto-immune disease) as time-independent covariates. Hb, hemoglobin; CI, confidential interval.

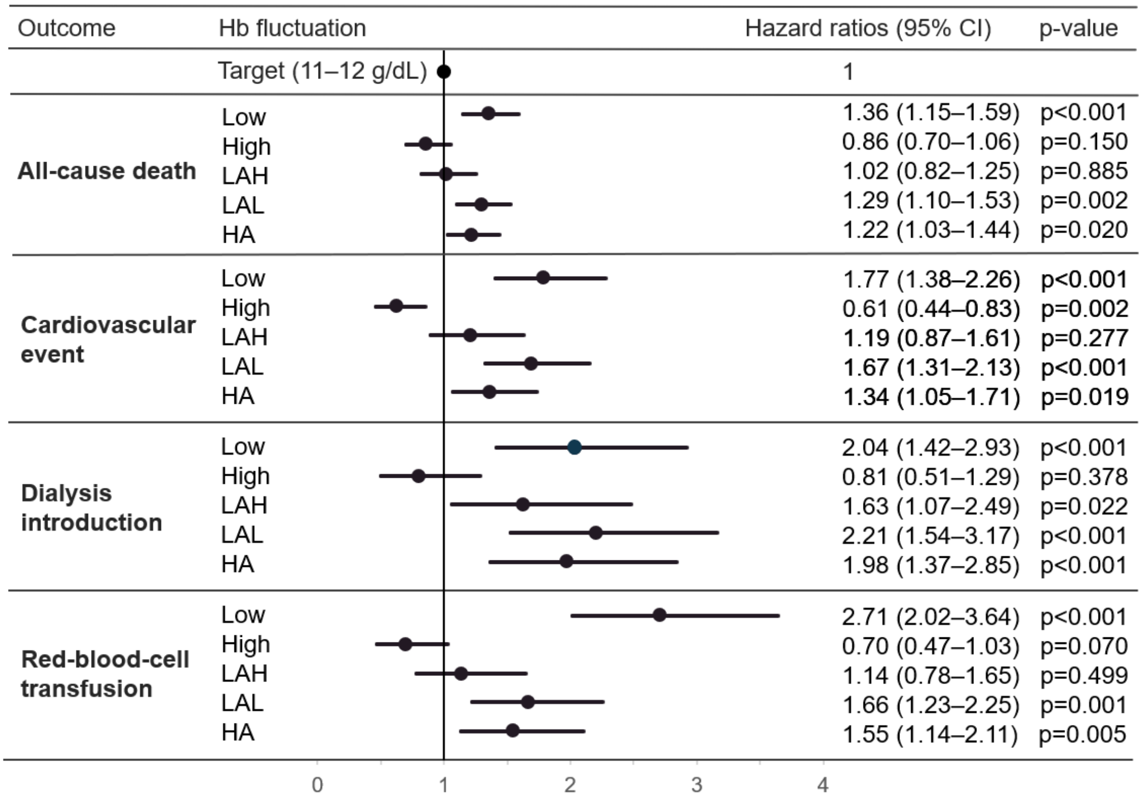

References

1. Andersen PK, Borgan Ø, Gill RD, et al. Statistical models based on counting processes. Springer: New York, NY, USA, 1993.

2. Kuragano T, Matsumura O, Matsuda A, et al. Association between hemoglobin variability, serum ferritin levels, and adverse events/mortality in maintenance hemodialysis patients. *Kidney Int.* 2014; 86: 845-854.

3. Boudville NC, Djurdjev O, Macdaugall IC, et al. Hemoglobin variability in non-dialysis chronic kidney disease: examining the association with mortality. *Clin J Am Soc Nephrol.* 2009; 4: 1176-1182.

4. Japanese Society of Nephrology. Essential points from evidence-based clinical practice guidelines for chronic kidney disease 2018. *Clin Exp Nephrol.* 2019; 23(1): 1-15.

5. Yamamoto H, Nishi S, Tomo T, et al. 2015 Japanese Society of Dialysis Therapy: guidelines for renal anemia in chronic kidney disease. *Renal Replacement Therapy.* 2017; **3**: 36.

6. von Elm E, Altman DG, Egger M, et al. The Strengthening the Reporting of Observational Studies in Epidemiology (STROBE) Statement: guidelines for reporting observational studies. Ann Int Med. 2007; 147(8): 573-577.
